# Neuroprotective Activity of Peripherally Administered Liver Growth Factor in a Rat Model of Parkinson’s Disease

**DOI:** 10.1371/journal.pone.0067771

**Published:** 2013-07-04

**Authors:** Rafael Gonzalo-Gobernado, Lucía Calatrava-Ferreras, Diana Reimers, Antonio Sánchez Herranz, Macarena Rodríguez-Serrano, Cristina Miranda, Adriano Jiménez-Escrig, Juan José Díaz-Gil, Eulalia Bazán

**Affiliations:** 1 Servicio de Neurobiología, Instituto Ramón y Cajal de Investigación Sanitaria, Madrid, Spain; 2 Servicio de Neurología, Hospital Universitario Ramón y Cajal, Madrid, Spain; 3 Instituto de Investigación Sanitaria Puerta de Hierro-Majadahonda, Madrid, Spain; Emory University, United States of America

## Abstract

Liver growth factor (LGF) is a hepatic mitogen purified some years ago that promotes proliferation of different cell types and the regeneration of damaged tissues, including brain tissue. Considering the possibility that LGF could be used as a therapeutic agent in Parkinson’s disease, we analyzed its potential neuroregenerative and/or neuroprotective activity when peripherally administered to unilaterally 6-hydroxydopamine (6-OHDA)-lesioned rats. For these studies, rats subjected to nigrostriatal lesions were treated intraperitoneally twice a week with LGF (5 microg/rat) for 3 weeks. Animals were sacrificed 4 weeks after the last LGF treatment. The results show that LGF stimulates sprouting of tyrosine hydroxylase-positive terminals and increases tyrosine hydroxylase and dopamine transporter expression, as well as dopamine levels in the denervated striatum of 6-OHDA-lesioned rats. In this structure, LGF activates microglia and raises tumor necrosis factor-alpha protein levels, which have been reported to have a role in neuroregeneration and neuroprotection. Besides, LGF stimulates the phosphorylation of MAPK/ERK1/2 and CREB, and regulates the expression of proteins which are critical for cell survival such as Bcl2 and Akt. Because LGF partially protects dopamine neurons from 6-OHDA neurotoxicity in the substantia nigra, and reduces motor deficits in these animals, we propose LGF as a novel factor that may be useful in the treatment of Parkinson’s disease.

## Introduction

Parkinson’s disease (PD) is a neurodegenerative disorder involving a progressive loss of dopaminergic (DA) neurons projecting from the substantia nigra (SN) to the striatum. The most widely used therapeutic approach is the administration of levodopa, but it loses effectiveness after several years of treatment. Neurotrophic factors are compounds that enhance the survival and differentiation of selected types of neurons, including DA neurons [1]. Liver growth factor (LGF) is a hepatic mitogen purified by Díaz-Gil and colleagues some years ago [2]. Following an in-depth chemical and immunological study, they demonstrated that LGF is an albumin–bilirubin complex, the concentration of which is nearly undetectable in sera from healthy humans or rats, but dramatically increases in the presence of hepatobiliary disorders or liver injury [3], [4]. Recent studies show that LGF promotes proliferation of different cell types [5]–[9] and the regeneration of damaged cells and tissues, including brain tissue. Thus, the intracerebral infusion of LGF stimulates the sprouting of DA terminals in the striatum of unilaterally 6-hydroxydopamine (6-OHDA)-lesioned rats [10], and promotes the expansion of neural precursors and the generation of new neurons in this experimental model of PD [11]. Moreover, its delivery into the brain enhances cell viability of grafted neural stem cells, and favors their differentiation to an endothelial-like phenotype [12].

The first targets of LGF in liver are portal vein endothelial cells [13], while DA sprouting and neurogenesis seem to be mediated by activated microglia/macrophages and reactive astrocytes [10], [11]. Besides, the mitogenic activity of LGF in rat liver is mediated by local and temporary up-regulation of tumor necrosis factor-alpha (TNF-alpha) [13], a cytokine synthesized and released by activated microglia [14], [15], which has recently been reported to have a role in neuroregeneration and neuroprotection [16]–[18].

Considering the possibility that LGF could be used as a therapeutic agent in PD, we analyze the potential neuroregenerative and/or neuroprotective activity of intraperitoneally administered LGF (IP-LGF) in a known model of PD in rats. Here we report that IP-LGF raises DA levels and stimulates the outgrowth of DA terminals in the striatum of unilaterally 6-hydroxydopamine (6-OHDA)-lesioned rats and protects DA neurons from 6-OHDA neurotoxicity. Moreover, IP-LGF reduces apomorphine-induced rotational behavior and improves motor peformace in these animals. In this study, we also show that IP-LGF regulates the expression of proteins that are critical for cell survival, and modulates the activity of both, the mitogen-activated protein kinase/extracellular signal-regulated kinase (MAPK/ERK1/2) and the phosphatidylinositol 3-kinase (PI3K)/Akt signal transduction pathways. The role of activated microglia and TNF-alpha in these LGF-mediated effects is also discussed.

## Materials and Methods

### LGF Purification

LGF was purified from serum of 5-week bile duct-ligated rats following a previously reported procedure [6]. LGF was quantitated by HPLC [19] and samples with the highest serum LGF concentrations were selected to proceed with the purification process, which involved three chromatography steps employing Sephadex G-150, DEAE-cellulose and hydroxylapatite. Purity, that is, the absence of other growth factors and/or contaminants in the LGF preparation, was also assessed according to standard criteria [2]–[4], [6], [19]. All LGF preparations showed a single band in sodium dodecyl sulfate polyacrylamide gel electrophoresis (SDS-PAGE). LGF preparations were lyophilized and kept at 4°C until use, at which time aliquots were dissolved in saline for intraperitonal injection.

### Ethics Statement

All procedures used in this work were in accordance with the European Union Council Directive. (86/609/EEC). The protocol was approved by the Committee on the Ethics of Animal Experiments of the Hospital “Ramón y Cajal” (animal facilities ES280790002001).

### Animals and 6-OHDA Lesion Surgery

A total of 114 female Sprague Dawley rats weighting 220–250 g were obtained from our animal facilities (Hospital Ramón y Cajal). The animals were housed in a temperature-controlled environment with 12 h light/dark cycles and access to food and water ad libitum. The intrastriatal injection of 6-OHDA is one of the most appropriate models to study early and late stages of PD [20]. Under isoflurane anesthesia, rats received four stereotaxic injections of 6-OHDA in the left striatum as previously described [21]. Using a 10-µl Hamilton syringe, 2.0 µl of 6-OHDA (3.5 µg/µl in 0.2 mg/ml L-ascorbate-saline) were injected into the left striatum as follows: zone 1, AP: +1.3; ML: −2.6 and DV: −5.0 (in mm with respect to bregma and dura; tooth bar at 0.0 mm); zone 2, AP: +0.4; ML: −3.0 and DV: −5.0; zone 3, AP: −0.4; ML: −4.2 and DV: −5.0; and zone 4, AP: −1.3; ML: −4.5 and DV: −5.0, according to the stereotaxic atlas of Paxinos and Watson (1997) [22]. The injection rate was 1 µl/min.

### Behavioral Testing

Apomorphine-induced rotational behavior is a good indicator of extensive lesions of the nigrostriatal pathway [20], and is considered a valuable behavioral index to test novel drugs against PD [23], [24]. At ten days following unilateral lesion of the nigrostriatal pathway, motor asymmetry was monitored over 15 min after subcutaneous injection of apomorphine (0.5 mg/kg diluted in 0.9% saline). At 6 weeks post-lesion (lesion control group) rats rotating at least 100 turns in 15 min [183±9 (n = 47) rotations in 15 min] were divided in two homogeneous groups, and selected for IP-LGF [188±11 (n = 23) rotations in 15 min] or vehicle [194±13 (n = 24) rotations in 15 min] administration. After initiation of treatment, rotation was monitored once a week until the end of the study period.

Motor performance was also analyzed using the rotarod test that is a useful drug-free procedure for evaluation of motor abilities in rat models of PD [25], [26]. Before 6-OHDA lesions were produced, rats received 3 independent training sessions in the rota-rod (PanLab S.L., Mod. LE 8500, Cornellá, Spain), with 4 1-minute evaluations at 4 to 40 rpm (accelerating rod). Those animals that withstood more than 1 minute at 4 to 40 rpm were selected for 6-OHDA lesions. Lesioned control rats which had mean latencies to fall on the accelerating rod of 16±2 s (n = 8) were selected for IP-LGF or vehicle administration. Starting 10 days after 6-OHDA lesion procedure, animals were monitored once a week until the end of the study period.

### LGF Administration

**Figure 1:**
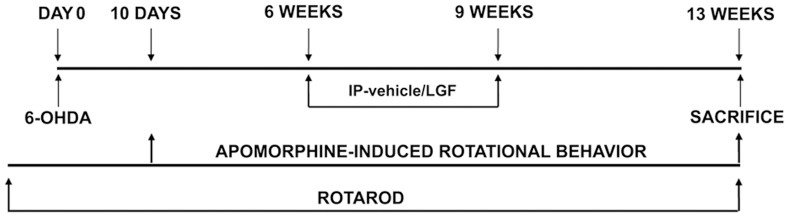
Representative scheme showing the methodological procedure followed for the repeated administration of vehicle and LGF to 6-OHDA-lesioned rats.

Six weeks after the unilateral 6-OHDA lesions were produced, rats received 2 weekly IP injections of saline (IP-vehicle) or LGF [5 µg/rat] (IP-LGF) for 3 weeks (Fig. 1A). This optimal dose of LGF has been used in different model systems using an identical o similar scheduled [5], [6], [8], [9]. This group of animals was sacrificed 4 weeks after the last treatment with saline or LGF (chronic treatment). A second group of rats received a single IP-vehicle or IP-LGF injection 13 weeks after 6-OHDA lesions were produced. These animals were sacrificed 24, 48, or 72 hours later (acute treatment).

### Tissue Processing

Thirteen weeks after the lesion with 6-OHDA the animals were perfused intracardially under deep anesthesia with 50 ml of isotonic saline, followed by 250 ml of 4% paraformaldehyde. Brains were postfixed in the same solution for 24 hr at 4°C, cryoprotected and frozen, before sectioning into 20-µm-thick coronal sections on a cryostat.

### Antibodies and Immunochemicals

The primary antibodies used in this study were: mouse anti-tyrosine hydroxylase (anti-TH, 1∶500; Chemicon International Inc., Temecula, CA), rabbit anti-proliferating cell nuclear antigen (PCNA, 1∶75; Santa Cruz Biotechnology Inc., Santa Cruz, CA), mouse anti-5-bromodeoxyuridine (BrdU, 1∶25; DakoCytomation, Denmark), rabbit anti-β-tubulin III (1∶500; BabCO, Richmond, CA), rabbit anti-glial fibrillary acidic protein (GFAP, 1∶200; DakoCytomation), mouse anti-neuronal nuclei (NeuN, 1∶1000; Chemicon International Inc), guinea pig anti-doublecortin (Dcx, 1∶3000; Chemicon International Inc.), mouse anti-OX6 (1∶250, AbD Serotec, Oxford, UK), rabbit anti-dopamine transporter (anti-DAT, 1∶50; Chemicon International Inc.), rabbit anti-phospho-CREB (ser 133) (1∶500; Millipore Ibérica S.A.U., Madrid, Spain), rabbit anti-TNF-alpha (1∶250, Santa Cruz Biotech), mouse anti Bcl2 (1∶25; Santa Cruz Biotech), rabbit anti-Bax (1∶250; Santa Cruz Biotech), peroxidase-labeled isolectin IB4 (Sigma Chemical Co, St Louis, MO), mouse-anti-ERK1/2 di(^Thr183yTyr185^P) (1∶500, Sigma Chemical). The secondary antibodies and other immunochemicals used were: Alexa Fluor-568 goat anti-mouse IgG, Alexa Fluor-488 donkey anti-rat IgG, and Alexa Fluor-488 goat anti-rabbit IgG (1∶400; all from Molecular Probes; Eugene, OR), fluorescein-conjugated goat anti-mouse IgG (1∶25; Jackson ImmunoResearch Laboratories Inc, West Grove, PA), Cy3-conjugated donkey anti-guinea pig IgG (1∶500, Jackson ImmunoResearch Laboratories Inc.), and rhodamine-conjugated goat anti-rabbit IgG (1∶100, Chemicon International Inc.).

### Immunohistochemistry and Morphometric Analysis

Tissue sections were mounted on positively charged slides (Dako REAL Capillary Gap microscope slides), treated with sodium acetate 10 mM, pH 6.0, at 95°C for 4 min, and preincubated with 5% normal goat serum (NGS) in Tris-buffered saline (TBS: 0.15 M NaCl and 0.1 M Tris HCl, pH 7.4)/0.1% Triton-X 100 for 30 min. Primary antibodies were applied for 24 hr at 4°C, and most of them were visualized using immunofluorescence procedures. The slides were coverslipped in a medium containing p-phenylenediamine and bisbenzimide (Hoechst 33342; Sigma) for detection of nuclei. The Bcl2 antibody was visualized using a biotinylated anti-mouse secondary antibody (DakoCytomation, Denmark), followed by incubation with streptavidin–biotin–peroxidase complex and finally, with diaminobenzidine (DAB) substrate–chromogen system (both from DakoCytomation). For double immunolabeling with BrdU, neural cell markers were detected prior to BrdU immunostaining. For detection of incorporated BrdU, sections were treated with 2N HCl at 37°C for 30 min and rinsed in TBS before blocking with NGS and primary antibody incubation.

For quantitative estimation of TH and DAT immunostaining in the striatum, measurements were performed in several coronal sections from 7 different rostrocaudal levels beginning at +1.6 mm relative to bregma (level 1), +1.00 mm (level 2), +0.2 mm (level 3), −0.3 mm (level 4), −0.92 mm (level 5), −1.4 mm (level 6) and −1.8 mm (level 7), using a ×10× objective and with the aid of the Computer Assisted Stereology Toolbox (CAST)® grid system (Olympus, Ballerup, Denmark). The area occupied by TH-positive fibers was expressed as a percentage of the total striatal cross-sectional area. TH/DAT and TNF-alpha/OX6 double inmunolabeled images were taken using a Nikon Eclipse Ti microscope coupled to a Nikon C1 confocal system.

The number of TH-positive cells in the SN pars compacta (SNpc) was assessed in 20-µm coronal sections from 2 different anteroposterior levels, separated by 140 µm, and establishing the first section at −5.3 mm relative to bregma with a 20× objective. The number of Bcl2-positive cells in the SNpc was assessed in previously TH stained coronal sections with a 10× objective. Images were taken with a Nikon Eclipse Ti microscope equipped with a Nikon DS-2MV camera using the scan large image function of Nikon NIS elements software to stitch widefield images encompassing the entire SN. The area occupied by the SNpc was delimitated using the TH inmunolabeling as a reference. Bcl2-positive cell counts in SNpc were expressed as Bcl2-positive cells/mm^2^.

### Western Blotting Protein Analysis

The striata and mesencephalon of 6-OHDA-lesioned rats that received IP-vehicle (n = 18) or IP-LGF, either once from 24 to 72 hours post-treatment (n = 21) or twice weekly for 3 weeks (n = 12), were removed and dissected following a previously described methodology [27]. Tissue was homogenized (1∶8, w/v) with homogenization buffer (20 mM Tris–HCl, pH 7.5∶140 mM potassium chloride; 5 mM magnesium acetate; 1 mM dithiothreitol, 2 mM benzamidine, 1 mM EDTA, 2 mM EGTA, 0.5% Triton X-100, 10 µg/ml pepstatin A, 10 µg/ml leupeptin and 10 µg/ml antipain; 20 mM sodium β-glycerophosphate; 20 mM sodium molybdate; 200 mM sodium orthovanadate). Homogenates were centrifuged at 11,000 g for 20 min, and proteins were processed for Western blot analysis to determine the relative levels of several proteins. The procedures were performed at 4°C and samples were kept at −80°C until use. Aliquots of 30 µg of protein were separated by electrophoresis on 10–15% SDS-polyacrylamide minigels and transferred to nitrocellulose filters. Membranes were soaked in blocking solution (0.1 M PBS and 5% dry skimmed milk, pH 7.4) and incubated with the following primary antibodies diluted in 0.1 M PBS and 1% dry skimmed milk, pH 7.4: mouse anti-Bcl2 (1∶400; Santa Cruz Biotechnology Inc., Burlingame, CA, USA), rabbit anti-Bax (1∶300; Santa Cruz Biotechnology Inc.), mouse anti-tyrosine hydroxylase (TH, 1∶5000; Chemicon International, Temecula, CA), rabbit anti-proliferating cell nuclear antigen (PCNA, 1∶1000; Santa Cruz Biotechnology, Santa Cruz, CA), rabbit anti-glial fibrillary acidic protein (GFAP, 1∶5000; DakoCytomation, Denmark), rabbit anti-Glut5 (1∶500, Abcam), mouse anti-OX6 (1∶1000, AbD Serotec, Oxford, UK), rabbit anti-DAT (1∶500; Chemicon), rabbit anti-phospho-CREB (ser 133) (1∶1000; Upstate), rabbit anti-TNF-alpha (1∶400; Santa Cruz Biotech), mouse-anti-ERK1/2 di(^Thr183yTyr185^P) (1∶5000, Sigma Chemical), mouse-anti-ERK1/2 (1∶10000, Sigma Chemical), rabbit anti-Akt (^Ser473^P) (1∶2000; Cell Signaling Technology, Beverly, MA, USA), rabbit anti-Akt (1∶2000; Cell Signaling Tecnology), rabbit anti-VMAT2 (1∶1000, Alpha Diagnostic International Inc., Texas, USA). After extensive washing in 0.05% PBS-Tween, membranes were incubated with the peroxidase-conjugated or alkaline-phosphatase-conjugated secondary antibodies diluted 1∶2000 in blocking solution. The membranes were developed with enhanced chemiluminescence Western blotting, following the manufacturer’s instructions (Amersham, Buckinghamshire, England), and were exposed to hyperfilm. Membranes were also immunolabeled for loading control using mouse anti-β actin (1∶5000; Sigma Aldrich) and anti-mouse IgG alkaline phosphatase-conjugated (1∶3000, Sigma Aldrich) and were developed with alkaline phosphatase reagent. The density of stained bands was scanned and quantified with the Image QuantTL software package and the data were normalized with respect to β-actin levels.

### Dopamine Detection by HPLC

Thirteen weeks after the lesion, the striata from 6-OHDA-lesioned rats that received IP-vehicle (n = 7) or IP-LGF (n = 4) were rapidly dissected, frozen in dry ice and stored at −80°C until analysis. The tissue was sonicated (VibraCell, level 2 for 30 seconds) in 8 volumes (w/v) of ice-cold 0.4 N perchloric acid (PCA) and then centrifuged for 20 min at 11,000 g and 4°C. Twenty µl of the supernatant was then used to determine DA levels, and the pellet was used for protein analysis. DA was measured according to Mena et al., [28]. Briefly, the supernatants were analyzed using an electrochemical detector equipped with a 5011A analytical cell and a 5021A conditioning cell (ESA Coulochem III, Chelmsford, MA). The voltage conditions were E1+400 mV, E2+50 mV. A five µm (150×4.6 mm) C18 column was used (ACE Aberdeen, Scotland). The mobile phase consisted of a 0.1 M citrate/acetate buffer pH 3.9 plus 10% methanol, 1M EDTA and 4.8 mM heptane sulfonic acid, and the flow rate was 1 ml/min.

### Data Analysis

Results are expressed as mean ± SEM of (n) independent animals. Statistical analyses for immunohistochemical and biochemical studies were performed using Student’s t-test, or one-way ANOVA followed by the Newman-Keuls multiple comparison test. For behavioral studies, a two-way ANOVA followed by Bonferroni multiple comparison test was used. Differences were considered significant when p≤0.05.

## Results

### Effects of LGF Treatment on TH Expression and DA Levels

**Figure 2:**
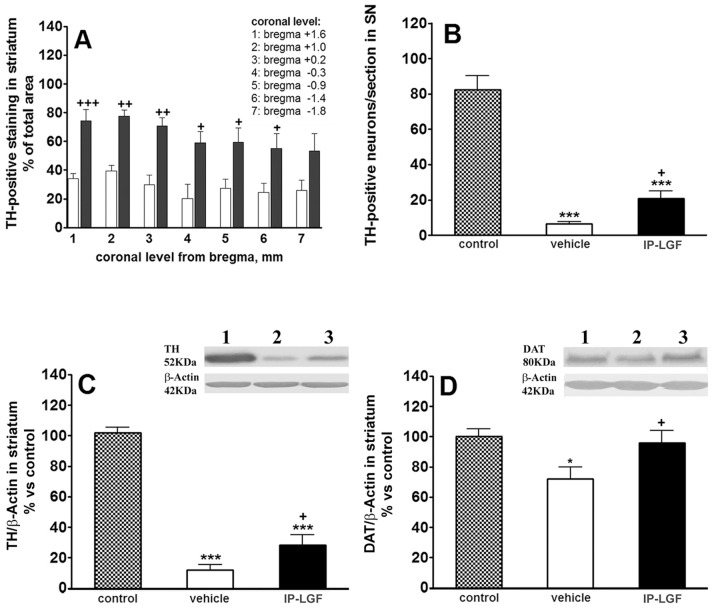
Liver growth factor stimulated TH and DAT protein expression in striatum and protected DA neurons in the substantia nigra of 6-OHDA-lesioned rats. Panel A shows TH-positive innervation in the striatum of lesioned rats treated with vehicle (A, white bars) or with LGF (A, black bars). Data in B represent the number of TH-positive neurons in the naïve (B, dotted bar) and lesioned substantia nigra (SN) of rats treated with vehicle (B, white bar) or LGF (B, black bar). Panels C and D show TH and DAT protein expression in the naïve (control, dotted bars) and lesioned striatum of rats receiving vehicle (white bars) or LGF (black bars). Results are represented as the mean ± SEM of n individual animals. T-tests were performed in A (n = 4–9. +p≤0.05, ++p≤0.01, and +++p≤0.001 vs vehicle). One way ANOVA were performed in B (p<0.0001; F_2, 24_ = 59.26, n = 8–11), C (p<0.0001; F_2, 41_ = 126.9, n = 11–17) and D (p = 0.0216; F_2, 25_ = 4.490, n = 5–12) followed by Newman-Keuls multiple comparison test (*p≤0.05 and ***p≤0.001 vs control. +p≤0.05 vs vehicle). C, D, lane 1: control striatum; C, D, lane 2: lesioned striatum of vehicle-treated rats; C, D, lane 3: lesioned striatum of LGF rats.

**Figure 3:**
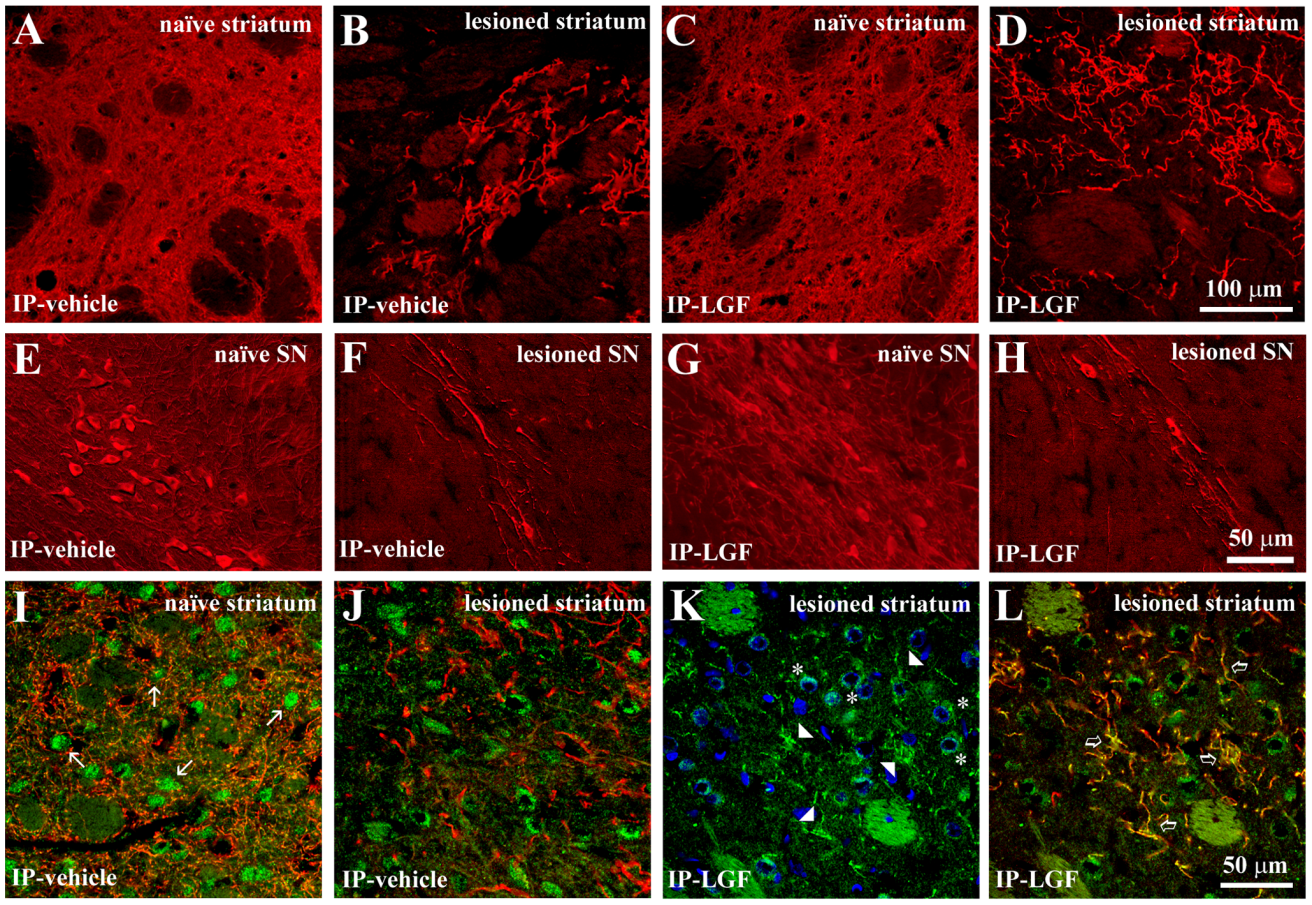
Liver growth factor modulated TH and DAT immunoreactivity in the striatum of 6-OHDA-lesioned rats. Panels A-D show TH immunostaining in the naïve striatum of IP-vehicle or LGF-treated rats (A, C red, respectively), and in the lesioned striatum of vehicle-treated rats (B, red), and rats receiving LGF (D, red). E, F, G and H show TH immunostaining in the naïve substantia nigra (SN) of vehicle (E, red) or LGF-treated rats (G, red), and in the lesioned SN of IP-vehicle (F, red) and IP-LGF-treated rats (H, red). Panels I, J and L show double immunostaining for DAT (green) and TH (red). K shows DAT immunostaining (green) and nuclei (blue). DAT immunoreactivity was reduced in the lesioned striatum of vehicle-treated rats (J, green), as compared with the naïve striatum of vehicle-treated rats (I, green) and with the lesioned striatum of the LGF group (K, green). Note how, in the naïve striatum of vehicle-treated, DAT immunostaining (I, green) is confined to small spots in the striatal parenchyma that probably represent neuronal cell bodies (white arrows in I). However, in the lesioned striatum of IP-LGF-treated rats, DAT immunoreactivity is distributed in neuronal cell bodies (asterisks in K) and in the striatal terminals (arrowheads in K). In addition, DAT is mainly expressed in the sprouting TH-positive terminals in the lesioned striatum of rats receiving LGF (L, yellow, clear arrows), while almost no TH/DAT co-labelling is observed in the lesioned striatum of IP-vehicle treated rats (J). Scale bar: A-D, 100 µm, and E-L, 50 µm.

Our previous studies in an experimental model of PD indicate that intracerebral administration of LGF stimulates the outgrowth of lesioned DA terminals. To verify the neuroregenerative activity exerted by LGF treatment *in vivo*, unilaterally 6-OHDA-lesioned rats were treated IP twice a week with vehicle or LGF (5 µg/rat) for 3 weeks. Animals were sacrificed 4 weeks after the last treatment, and immunohistochemical analysis of coronal sections of the striatum and the SN was performed. As shown in Fig. 2A, the 6-OHDA-lesioned striatum of animals treated with vehicle showed TH-positive innervation in 35% of the structure. By contrast, in the LGF-treated group, more than 60% of the lesioned striatum presented TH-positive innervation in all the coronal sections analyzed (Fig. 2A). Although the effect of LGF on TH-positive innervation was seen at different levels of the striatum, it was most marked in the dorsal and central regions of the structure (Fig. 2A and Fig. 3A–D). Western blot analysis also showed that TH protein expression was partially restored in the 6-OHDA-lesioned striatum of LGF-treated rats as compared with animals receiving vehicle (Fig. 2C). Besides, IP-LGF raised DA levels from 2.9±0.6 to 4.9±0.4 ng DA/mg protein in this structure [t_ = _2,44, df = 8, p = 0.0405].

In the naïve striatum of 6-OHDA-lesioned rats, LGF treatment did not significantly affected TH-positive innervation (Fig. 3A, C), TH protein expression [107±6% of IP-vehicle striatum], and DA levels [66±19 and 88±4 ng DA/mg protein in IP-vehicle and IP-LGF treated rats, respectively].

In the SNpc of vehicle-treated rats, a few DA neurons remained in the 6-OHDA-lesioned side as compared with their naïve side (Fig. 2B and Fig. 3J, K), and with the lesioned SNpc at 6 weeks post-lesion [15±1.3 (n = 9) and 6.5±1.3 (n = 8) TH-positive neurons/section at 6 and 13 weeks post-lesion, respectively, t_ = _4.589, df = 15, p = 0.0004]. LGF treatment significantly increased the total number of TH-positive neurons when compared with vehicle-treated animals at 13 weeks post-lesion in two coronal sections of the structure analyzed (Fig. 2B, and 3E–F). By contrast, IP-LGF did not affect total number of TH-positive neurons in the naïve SNpc of 6-OHDA-lesioned rats [82±7.9 (n = 8) and 103±9.9 (n = 11) TH-positive neurons/section in the naïve striatum of IP-vehicle and IP-LGF treated rats, respectively].

Since we have previously demonstrated the neurogenic activity of LGF *in vivo* [11], we considered that LGF treatment could induce the generation of DA neurons in the lesioned striatum and/or SNpc. Double immunostaining showed no TH-positive/BrdU-positive cells in the naïve and lesioned striatum of vehicle and LGF-treated animals. Moreover, none of the TH-positive neurons in the SN presented BrdU-positive nuclei (data not shown), suggesting the lack of neurogenic activity in the case of IP administration of LGF to 6-OHDA-lesioned rats.

### LGF Treatment Enhances DAT Protein Expression in the DA-depleted Striatum of 6-OHDA-lesioned Rats

Other DAergic markers such as DAT and VMAT2 were also analyzed in the striatum and mesencephalon of 6-OHDA-lesioned rats. As shown in Fig. 2D, DAT protein expression was significantly lower in the DA-depleted striatum of vehicle-treated rats as compared with the contralateral side of the structure. The 3-week treatment with LGF restored DAT protein expression to levels similar to those observed in the naïve striatum of the vehicle group (Fig. 2D).

The immunohistochemical analysis of DAT gave similar results. As expected, DAT immunostaining was lower in the DA-depleted striatum of vehicle-treated rats as compared with the unlesioned side of the structure (Fig. 3I–J). Moreover, DAT immunoreactivity was higher in the lesioned striatum of animals receiving IP-LGF for 3 weeks (Fig. 3K). LGF treatment also affected the distribution of DAT. Thus, in the naïve and lesioned striatum of IP-vehicle-treated rats DAT immunostaining was localized in small spots distributed in the striatal parenchyma that seem to correspond to neuronal cell bodies (Fig. 3I–J). However, in the lesioned striatum of IP-LGF-treated rats, DAT immunoreactivity was distributed in neuronal cell bodies, and in the sprouting TH-positive terminals (Fig. 3K–L).

Neither DA depletion nor the 3-weeks treatment with LGF affected VMAT2 protein expression in the striatum. On the other hand, LGF treatment did not modify DAT and VMAT2 protein expression in the mesencephalon of 6-OHDA-lesioned rats (data not shown).

### LGF Treatment Reduces Motor Deficits in 6-OHDA-lesioned Rats

**Figure 4:**
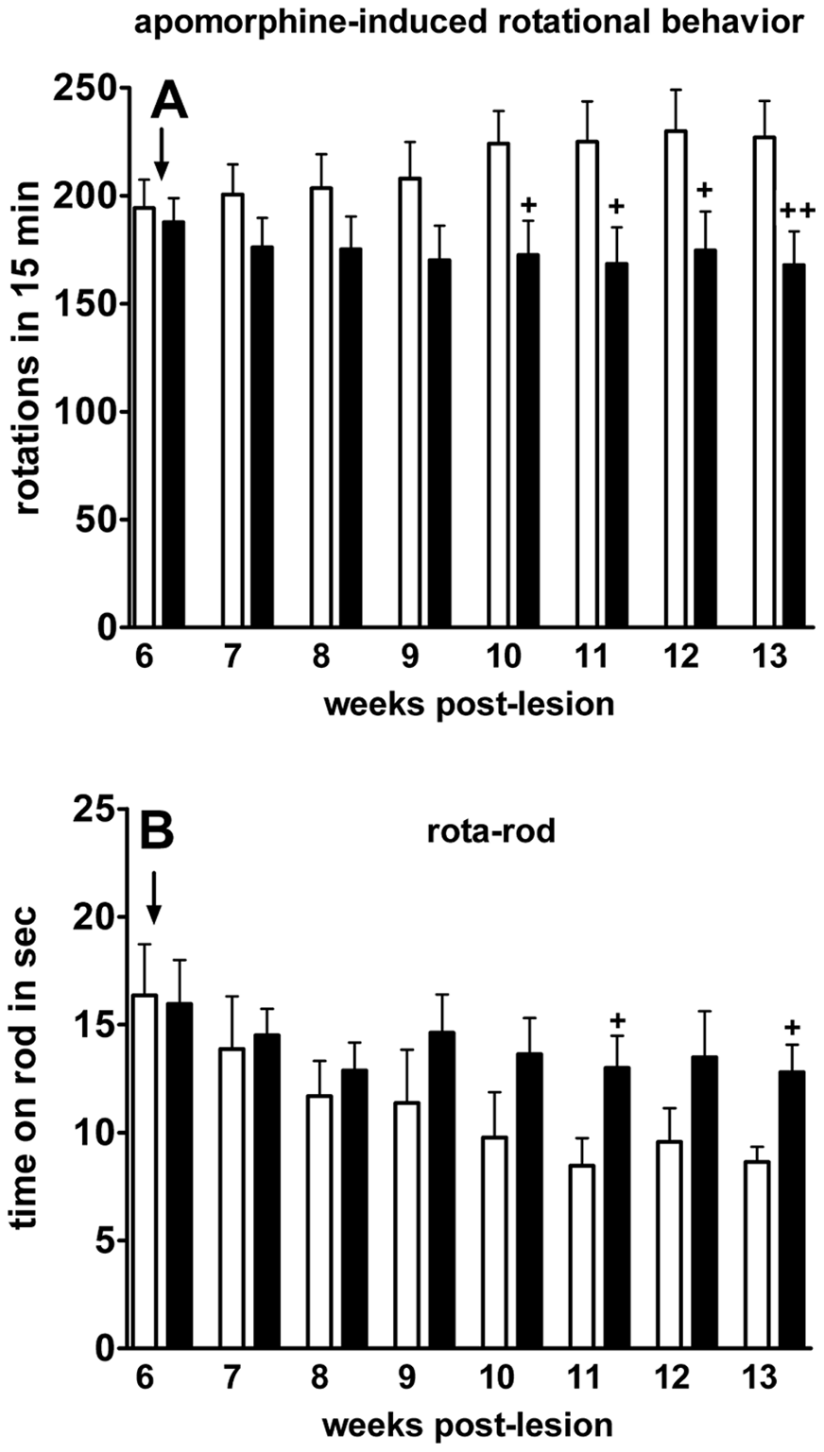
LGF ameliorated motor deficits in 6-OHDA-lesioned rats. Between 10 and 13 weeks post-lesion, the number of apomorphine-induced contralateral rotations was significantly reduced in 6-OHDA-lesioned rats receiving LGF (A, black bars), as compared with rats treated with vehicle (A, white bars). Results represent the mean ± SEM of 23 to 24 individual rats. Motor performance, as assessed by the rotarod test, was reduced in lesioned rats receiving vehicle (B, white bars). Note how, between 11 and 13 weeks post-lesion, LGF significantly improved this parameter (B, black bars). Results represent the mean ± SEM of 4 individual animals. Two-way ANOVA was performed in A (p<0.0001; F_1, 351_ = 26.08) and B (p = 0.0166; F_1, 48_ = 6.16) followed by Bonferroni multiple comparison test (+p≤0.05, ++p≤0.01 vs vehicle). Start of treatments (↓).

To determine whether IP-LGF was functional *in vivo*, two motor behavioral tests were performed. Apomorphine-induced rotations were evaluated in rats with unilateral 6-OHDA lesions once a week, before, during, and after the treatment period. As demonstrated in Fig. 4A, 4 weeks after LGF treatment had begun, the LGF-treated group of animals showed a significant reduction in apomorphine-induced rotational behavior as compared with vehicle-treated rats. Because the reduction in rotational behavior persisted for at least 3 additional weeks (Fig. 4A), our results suggest that IP-LGF treatment is able to improve motor deficit in 6-OHDA-lesioned rats in apomorphine rotation test.

We have also analyzed the effects of LGF on motor abilities using the rotarod test. Motor performance of naïve rats was relatively stable over repeated tests, resulting in mean latencies to fall on the accelerating rod of approximately 56.47±2 s (n = 8). As shown in Fig. 4B, rats with unilateral 6-OHDA lesions showed a progressive impairment that reached a plateau at 10 weeks post-lesion. This effect was not observed in the LGF-treated group of animals. Thus, in this experimental group motor performance was stable between 6 and 13 weeks post-lesion (Fig. 4B). Moreover, between 5 and 7 weeks after LGF treatment had begun their motor behavior was significantly improved, as compared with 6-OHDA-lesioned rats receiving vehicle (Fig. 4B).

### LGF Activates Microglia and Regulates TNF-alpha Protein Expression in the Striatum of 6-OHDA-lesioned Rats

**Figure 5:**
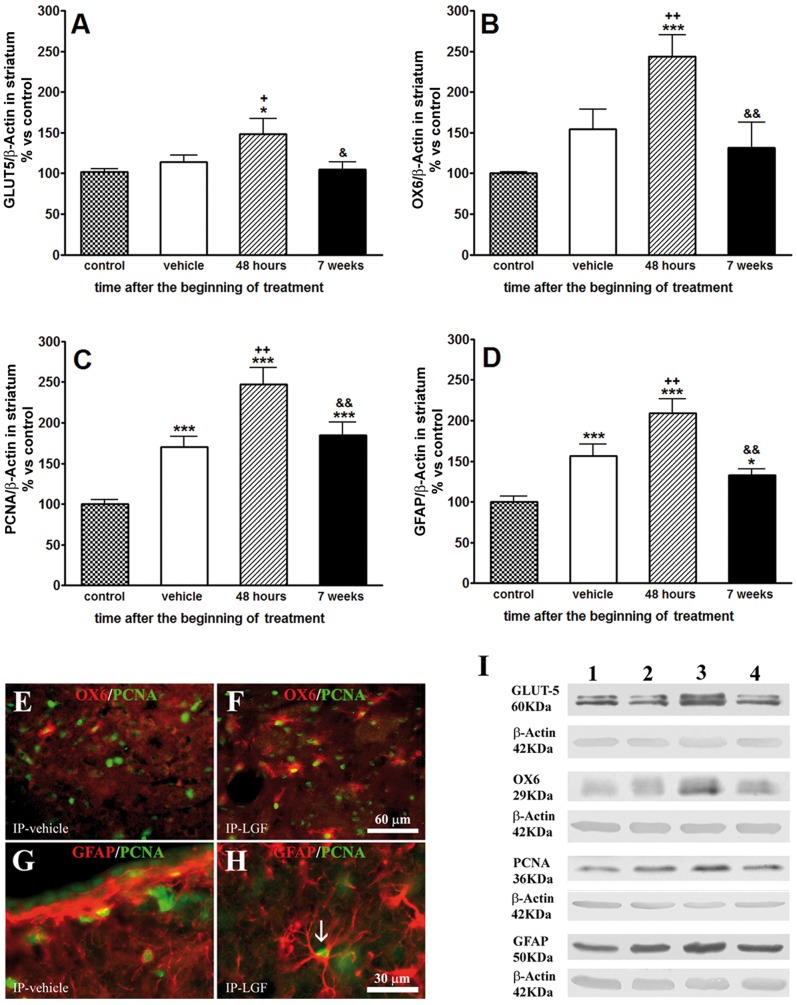
Liver growth factor activated glial cells in the striatum of 6-OHDA-lesioned rats. Panels A, B and D show the effect of IP-LGF treatment on proteins expressed by glial cells. Forty-eight hours after the administration of a single injection of LGF (A, B and D, lined bars), GLUT5, OX6, and GFAP protein levels were significantly higher than those observed in control (A, B and D, dotted bars) or in the lesioned striatum of vehicle-treated rats (A, B and D, white bars). Note how the chronic administration of LGF (A, B and D, black bars) significantly reduced the abovementioned parameters. Panel C shows the effect of IP-LGF administration on PCNA protein expression. Results are expressed as percentage of control (naïve striatum of IP-vehicle treated rats at 13 weeks post-lesion), and represent the mean ± SEM of n individual rats. One way ANOVA were performed in A (p = 0.0149; F_3, 47_ = 3.868, n = 7–18), B (p = 0.006; F_3, 41_ = 7.435, n = 7–12), C (p<0.0001; F_3, 48_ = 21.66, n = 7–18) and D (p<0.0001; F_3, 48_ = 12.75, n = 7–18) followed by followed by Newman-Keuls multiple comparison test (*p≤0.01, and ***p≤0.001 vs control. +p≤0.05, and ++p≤0.01 vs vehicle. &p≤0.05, and &&p≤0.01 vs 48 hours post-LGF). Panels E and F show double immunostaining for OX6 (red) and PCNA (green) in the lesioned striatum of rats receiving a single injection of vehicle or LGF. G and H show the immunoreactivity for GFAP (red) and PCNA (green) in the striatum of IP-vehicle and IP-LGF-treated rats. Note how, 48 hours after the administration of LGF, many OX6-positive cells were PCNA-positive too, and how LGF promoted cell body hypertrophy of GFAP-positive cells (H, white arrow). Scale bar: E-F, 60 µm, and G-H, 30 µm. Panel I shows representative blots for GLUT5, OX6, PCNA, and GFAP. Lane 1: control striatum; lane 2: lesioned striatum of vehicle rats; lane 3: lesioned striatum of 48-hour LGF-treated rats; lane 4: lesioned striatum of chronic LGF-treated rats.

Our previous studies suggest that glial cells could mediate the neuroregenerative and neurogenic effects promoted by the intracerebral administration of LGF in 6-OHDA-lesioned rats [10], [11]. To determine whether IP-LGF could stimulate microglia, we analyzed several parameters that are associated with the activation of this cell type. The levels of GLUT5, a glucose transporter expressed by microglia, were significantly raised in the 6-OHDA-lesioned striatum of rats receiving a single injection of LGF and sacrificed 48 hours later (Fig. 5A, I). The anti-OX6 antibody recognizes a histocompatiblility Class II antigen expressed by activated microglia. As shown in Fig. 5B, OX6 levels were enhanced in the DA-depleted striatum of animals receiving a single injection of LGF, as compared with the naïve and the lesioned striatum of the IP-vehicle-treated group. Similarly, 48 hours after administration of a single treatment, OX6 immunoreactivity was higher in the striatal parenchyma of IP-LGF-treated rats, as compared with those animals receiving vehicle (Fig. 5E, F).

Proliferation is part of the activation response of microglia. Western blot analysis showed that PCNA protein expression was increased 1.6-fold in the 6-OHDA-lesioned striatum of vehicle-treated rats as compared with the contralateral side of the structure. Forty-eight hours after the administration of LGF, PCNA levels were significantly raised as compared with the naïve and the 6-OHDA-lesioned striatum of IP-vehicle-treated rats (Fig. 5C, I). To determine whether IP-LGF promoted the proliferation of microglia, double immunostaining for PCNA and OX6 was performed. In the DA-depleted striatum of 6-OHDA-lesioned rats receiving IP-vehicle, a small population of PCNA-positive cells was immunoreactive for anti-OX6 (Fig. 5E). Forty-eight hours after IP-LGF treatment had begun, most of the PCNA-positive cells were also OX6-positive (Fig. 5F).

**Figure 6:**
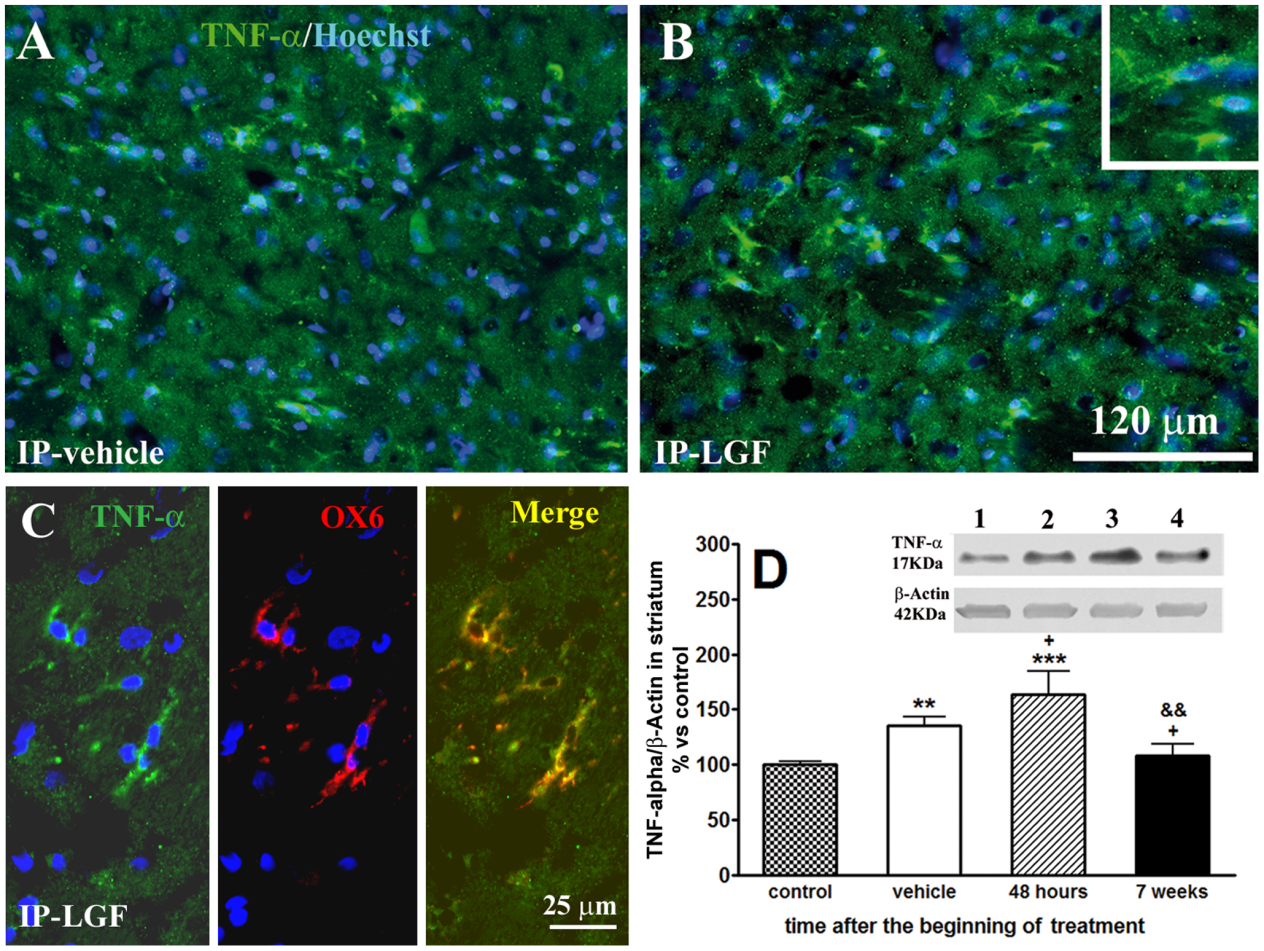
Liver growth factor elicited TNF-alpha expression in the striatum of 6-OHDA-lesioned rats. A and B show TNF-alpha (TNF-α) immunoreactivity in the lesioned striatum of vehicle-treated rats (A, green) or 48 hours after the administration of a single injection of LGF (B, green). Note how TNF-α-positive cells in the striatum exhibit the morphology of microglia (B, inset). Panel C shows TNF-α (green) and OX6 (red) immunolabeling in LGF treated rats. Note how, in this experimental group of animals, OX6-positive cells co-expressed TNF-α (C, merge, yellow). Scale bar: A-B, 120 µm, and C, 25 µm. D shows how, 48 hours after the administration of a single injection of LGF, TNF-α protein expression was significantly increased (D, lined bar), as compared with control (D, control, dotted bar), and vehicle-treated rats (D, white bar). Chronic administration of LGF (D, black bar) restored TNF-α protein levels to control values. Results are expressed as percentage of control (naïve striatum of IP-vehicle treated rats at 13 weeks post-lesion), and represent the mean ± SEM of 7 to 18 individual rats. One way ANOVA was performed in D (p<0.0001; F_3, 46_ = 9.057, n = 7–18) followed by Newman-Keuls multiple comparison test (**p≤0.01 and ***p≤0.001 vs control. +p≤0.05 vs vehicle. &&p≤0.01 vs 48 hours post-LGF). Lane 1: control striatum; lane 2: lesioned striatum of vehicle rats; lane 3: lesioned striatum of 48-hour LGF-treated rats; lane 4: lesioned striatum of chronic LGF-treated rats.

TNF-alpha is a cytokine synthesized and released by activated microglia whose upregulation mediates LGF activity in rat liver [13]. Western blot analysis showed that, in the DA-depleted striatum of rats receiving IP-vehicle, TNF-alpha levels were about 1.5-fold higher than those found in their naïve striatum (Fig. 6D). Forty-eight hours after the beginning of LGF treatment, TNF-alpha levels were significantly higher than those found in the lesioned striatum of rats receiving IP-vehicle (Fig. 6D). The immunohistochemical analysis of TNF-alpha gave similar results. Thus, the DA-depleted striatum of IP-LGF-treated rats showed a higher number of TNF-alpha-positive cells than the striatum of 6-OHDA-lesioned rats treated with vehicle (6A, B). At this experimental time, TNF-alpha immunoreactive cells were OX6-positive also (Fig. 6C) and exhibited the morphology of microglia (Fig. 6B, inset), indicating that LGF stimulates the synthesis of TNF-alpha in this cell type.

Reactive astrocytes could participate in the neuroregenerative activity promoted by LGF in 6-OHDA-lesioned rats. GFAP is a protein expressed by astrocytes, and its over-expression is a feature of glial reactivity. In the DA-depleted striatum of vehicle-treated rats, GFAP protein expression was increased 1.7-fold as compared with the unlesioned side of the structure. Forty-eight hours after the beginning of the treatment, GFAP levels were significantly higher in the 6-OHDA-lesioned striatum of animals receiving a single LGF injection than those found in the lesioned striatum of animals receiving IP-vehicle (Fig. 5D, I). At this experimental time, GFAP-positive cells in the striatal parenchyma of rats receiving IP-LGF showed cell body hypertrophy as compared with the IP-vehicle group (Fig. 5G, H). However, only a few PCNA−/GFAP-positive cells were observed in the lesioned striatum of both the IP-vehicle (Fig. 5G) and the IP-LGF treated animals (Fig. 5H).

We also analyzed the abovementioned parameters in the striatum of 6-OHDA-lesioned rats that received 2 IP-LGF injections twice a week for 3 weeks. The chronic administration of the factor reduced GLUT5, OX6, PCNA, GFAP, and TNF-alpha protein levels to those observed in the naïve (Fig. 5A, B and Fig. 6D) and the DA-depleted striatum of the IP-vehicle group (Fig. 5C, D). Altogether, our results indicate that the activation of glial cells in the DA-depleted striatum of 6-OHDA-lesioned rats is an early and transitory event in the action of LGF.

In the mesencephalon of 6-OHDA-lesioned rats, neither PCNA, nor TNF-alpha, GLUT-5, or GFAP protein expression was affected by LGF treatment at any post-treatment time analyzed. However, OX6 expression was reduced 0.62-fold in the lesioned mesencephalon of rats that received LGF for 3 weeks [t = 2.61, df = 19, p = 0.0172 vs the lesioned mesencephalon of IP-vehicle], suggesting a possible long-term antiinflammatory effect of LGF in the damaged SN.

### LGF Treatment Stimulates the Phosphorylation of MAPK/ERK_1/2_ and CREB in the Striatum of 6-OHDA-lesioned Rats

**Figure 7:**
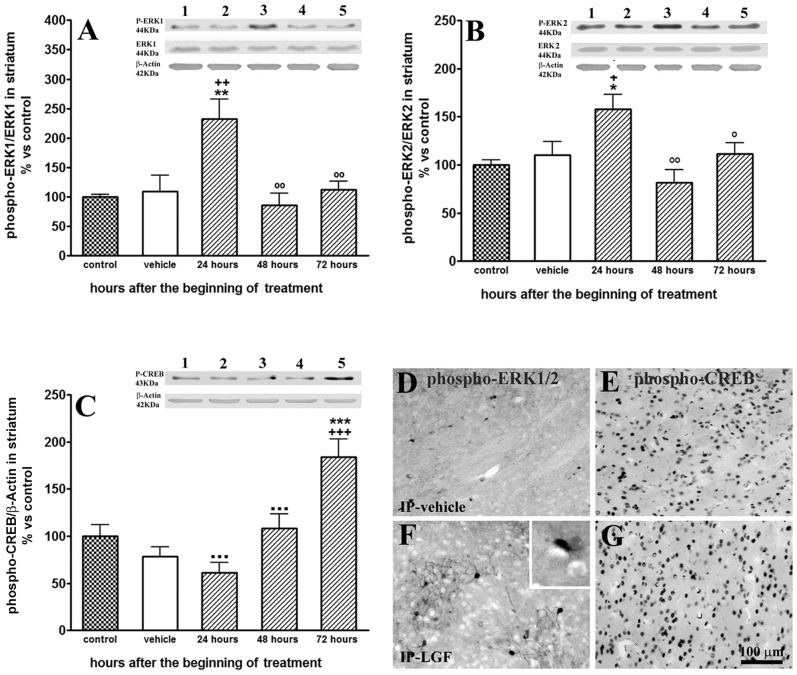
Liver growth factor activated the MAPK/ERK1/2 signalling pathway and elicited the phosphorylation of CREB in the striatum of 6-OHDA-lesioned rats. A single injection of LGF promoted a transient increase in the phosphorylation of ERK1/2, which was observed 24 hours after the administration of the factor (A, B, lined bars). Moreover, 72 hours after LGF treatment, phospho-CREB levels were significantly raised (C, lined bars), as compared with control (C, dotted bar) and vehicle-treated rats (C, white bars). Lane 1: control; lane 2: lesioned striatum of vehicle rats; lane 3: lesioned striatum of 24-hour LGF-treated rats; lane 4: lesioned striatum of 48-hour LGF-treated rats; lane 5: lesioned striatum of 72-hour LGF-treated rats. Results are expressed as percentage of control (naïve striatum of IP-vehicle treated rats at 13 weeks post-lesion), and represent the mean ± SEM of n individual rats. One way ANOVA were performed in A (p = 0.0006; F_4, 28_ = 6.791, n = 6–7), B (p = 0.0028; F_4, 29_ = 5.178, n = 6–7) and C (p<0.0001; F_4, 29_ = 11.58, n = 6–8), followed by Newman-Keuls multiple comparison test (*p≤0.05, **p≤0.01, and ***p≤0.001 vs control. +p≤0.05, ++p≤0.01, and +++p≤0.001 vs vehicle. °p≤0.05, °°p≤0.01 vs 24 hours post-LGF. ···p≤0.001 vs 72 hours post-LGF). Panels D-G show the immunohistochemistry for phospho-ERK1/2 (D and F) and phospho-CREB (E and G) in the lesioned striatum of vehicle (D and E), 24-hours LGF-treated rats (F), and 72-hours LGF-treated rats (G). Note how IP-LGF increased phospho-ERK1/2 (F) and phospho-CREB (G) immunostaining, and how phospho-ERK1/2-positive cells exhibit the morphology of ramified glia (F, inset). Scale bar: 100 µm.

The mitogen-activated protein kinase/extracellular signal-regulated kinase (MAPK/ERK1/2) signal transduction pathway regulates a variety of cellular activities including proliferation, survival and differentiation [29]. To determine whether this signaling pathway was activated by IP-LGF, Western blot analyses were performed in the striatum and mesencephalon of 6-OHDA-lesioned rats treated with a single LGF injection. In the striatum, the expressions of phospho-ERK_1/2_ and ERK_1/2_ were not affected by the lesion with 6-OHDA. Twenty-four hours after LGF administration, the expressions of phospho-ERK1 and phospho-ERK2 in the DA-depleted striatum were increased 2.7-fold [t_ = _2.837, df = 10, p = 0.0176] and 1.6-fold [t_ = _2.637, df = 12, p = 0.0217], respectively, as compared with the lesioned striatum of 6-OHDA-lesioned rats receiving vehicle. Because LGF treatment did not affect ERK_1/2_ levels, the ratios of phospho-ERK_1_/ERK_1_ (Fig. 7A) and phosphoERK_2_/ERK_2_ (Fig. 7B) were also significantly raised at this experimental time. The immunohistochemical analysis of phospho-ERK_1/2_ confirmed these results. Thus, the DA-depleted striatum of the IP-LGF-treated rats showed higher phospho-ERK_1/2_ immunoreactivity than the striatum of the IP-vehicle animals (Fig. 7D, F). Moreover, some of the phospho-ERK_1/2_ immunopositive cells in the IP-LGF group exhibited the morphology of ramified glial cells (Fig. 7F, inset).

The increase in phospho-ERK_1/2_ observed 24 hours after IP-LGF treatment was not maintained 48 or 72 hours later (Fig. 7A, B), indicating that the activation of ERK_1_/ERK_2_ MAPKs in the DA-depleted striatum of 6-OHDA-lesioned rats is an early event in the action of LGF. On the other hand, neither phospho-ERK1/2 nor ERK1/2 levels were affected in the mesencephalon of 6-OHDA-lesioned rats treated with LGF at any post-treatment time analyzed (data not shown).

Cyclic AMP response-element binding protein (CREB) is a transcription factor involved in neuronal cell survival and differentiation; its phosphorylation at Ser133, via the stimulation of the MAPK/ERK_1/2_ and other signaling pathways, is critical for its activation [30]–[34]. To investigate whether CREB could be a point of convergence mediating the effects of LGF, Western blot analyses were performed in the striatum and mesencephalon of 6-OHDA-lesioned rats treated with a single LGF injection. In the DA-depleted striatum, LGF promoted the activation of CREB. Thus, 72 hours after the administration of the factor, phospho-CREB protein expression was significantly raised as compared with the naïve, and the lesioned striatum of IP-vehicle-treated rats (Fig. 7C). By contrast, phospho-CREB levels in the lesioned mesencephalon were not affected by IP-LGF treatment at any experimental time studied (data not shown). The immunohistochemical analysis of phospho-CREB gave similar results. Thus, the lesioned striatal parenchyma of IP-LGF-treated rats showed higher phospho-CREB immunoreactivity than the striatum of the IP-vehicle animals (Fig. 7E, G). However, no differences for phospho-CREB immunostaining were observed between the lesioned mesencephalon of IP-vehicle and IP-LGF-treated rats.

### LGF Modulates Akt and Bcl2 Protein Expression in the Striatum and Mesencephalon of 6-OHDA-lesioned Rats

**Figure 8:**
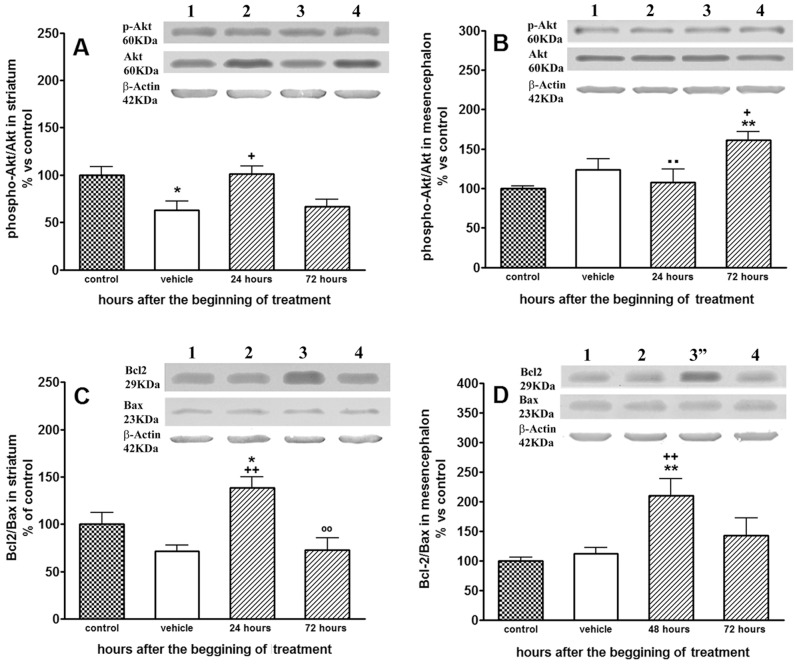
Liver growth factor modulated the expression of phospho-Akt and Bcl2 in the striatum and mesencephalon of 6-OHDA-lesioned rats. As shown in A, the decrease in phospho-Akt/Akt ratio observed in vehicle-treated rats (A, white bar) was prevented 24 hours after the administration of a single injection of LGF (A, lined bar). In the lesioned mesencephalon, the phospho-Akt/Akt ratio was significantly increased 72 hours after the beginning of LGF treatment (B, lined bars), as compared with control (B, dotted bar), and the lesioned mesencephalon of vehicle-treated rats (B, white bar). Panels C and D show Bcl2/Bax ratios in the lesioned striatum (C) and mesencephalon (D) of 6-OHDA-lesioned rats. Note how a single injection of LGF promoted a transitory, but significant, increase in Bcl2/Bax ratio in both structures. Lane 1: control; lane 2: vehicle-treated rats; lane 3∶24-hour LGF-treated rats; lane 3″: lesioned mesencephalon of 48-hour LGF-treated rats; lane 4∶72-hour LGF-treated rats. Results are expressed as percentage of control (naïve striatum and mesencephalon of IP-vehicle treated rats at 13 weeks post-lesion), and represent the mean ± SEM of n individual rats. One way ANOVA were performed in A (p = 0.0064; F_3, 26_ = 5.135, n = 6–11), B (p = 0.014; F_3, 22_ = 4.426, n = 6–7), C (p = 0.0009; F_3, 22_ = 8.003, n = 6–7) and D (p = 0.0047; F_3, 23_ = 5.651, n = 6–7) followed by Newman-Keuls multiple comparison test (*p≤0.05 and **p≤0.01 vs control. +p≤0.05 and ++p≤0.01 vs vehicle. °°p≤0.01 vs 24 hours post IP-LGF. ··p≤0.01 vs 72 hours post-LGF).

The protein Akt is a key downstream effector of the PI3K/Akt signaling pathway. In the DA-depleted striatum of IP-vehicle-treated rats Akt protein expression was 1.24±0.7-fold higher than in the unlesioned side of the structure [t_ = _2.867, df = 16, p = 0.0112 vs the naïve striatum of IP-vehicle treated rats] while phospho-Akt levels remained unchanged. Twenty-four hours after the administration of LGF, Akt levels were restored in the lesioned striatum, reaching values similar to those found in the naïve striatum of IP-vehicle-treated rats. IP-LGF treatment did not affect phospho-Akt levels, but significantly increased the ratio phospho-Akt/Akt by 1.6±0.13-fold (Fig. 8A). Similar results were observed in the lesioned mesencenphalon 72 hours after the administration of a single LGF injection. Thus, LGF treatment reduced Akt levels to 70±10% of control values [One way ANOVA p = 0,0027; F_2, 18_ = 4.183, followed by Newman-Keuls multiple comparison test, p≤0.05 vs the naïve and the lesioned mesencephalon of IP-vehicle treated rats, n = 7], and increased the phospho-Akt/Akt ratio by 1.6±0.1-fold (Fig. 8B). Altogether, these results suggest that IP-LGF treatment could modulate the activity of the PI3K/Akt signaling pathway in the striatum and mesencephalon of 6-OHDA-lesioned rats.

Bcl2 is an anti-apoptotic protein which expression can be regulated by different signaling pathways [35]–[37]. Twenty-four hours after the administration of a single LGF injection, Bcl2 protein expression was raised by 2.2±0.6-fold in the lesioned striatum [One way ANOVA p = 0,0259; F_2, 15_ = 4.706, followed by Newman-Keuls multiple comparison test, p≤0.05 vs the naïve and the lesioned striatum of IP-vehicle treated rats, n = 5–7 independent rats]. LGF treatment did not affect the levels of the pro-apoptotic protein Bax at this experimental time, so the Bcl2/Bax ratio in the DA-depleted striatum of IP-LGF-treated rats was significantly higher than that observed in the naïve and in the lesioned striatum of rats receiving IP-vehicle (Fig. 8C). Similarly, Bcl2 protein expression and the Bcl2/Bax ratio were significantly increased in the lesioned mesencephalon of IP-LGF-treated rats, but the effect was observed 48 hours after the administration of the factor (Fig. 8D). Neither the striatum nor the mesencephalon of IP-LGF-treated rats showed significant changes in the Bcl2/Bax ratio 72 hours after treatment (Fig. 8C, D).

**Figure 9:**
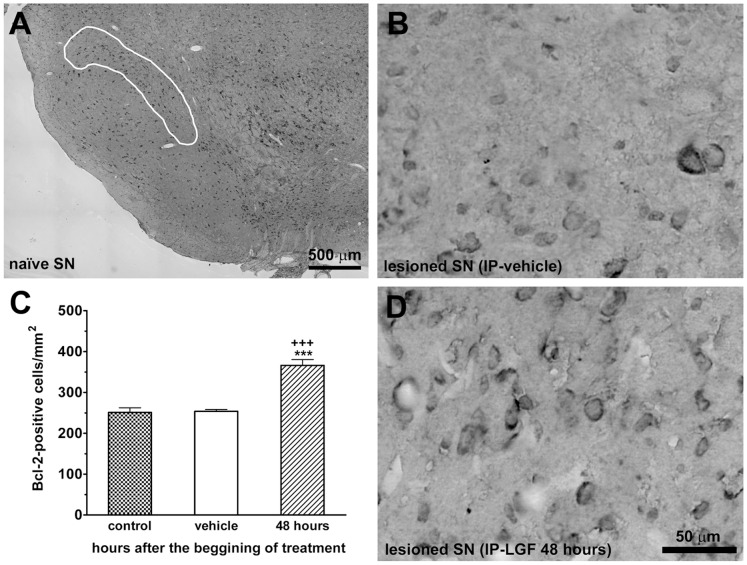
Liver growth factor modulated Bcl2 immunoreactivity in the substantia nigra of 6-OHDA-lesioned rats. Panels A, B, and D show Bcl2 immunostaining in the substantia nigra pars compacta (SNpc) of naïve rats (A), and in the lesioned SNpc of vehicle-treated rats (B), and 48 hours after receiving one single IP injection of LGF (D). The SNpc was assessed in previously TH stained coronal sections as described in the methods section (A, SNpc area delimited in white). Scale bar: A, 500 µm, and B, D, 50 µm. Data in C represent the number of Bcl2-positive cells in the naïve SN of IP-vehicle treated rats at 13 weeks post-lesion (control, dotted bar) and lesioned SN of rats treated with vehicle (white bar) or LGF for 48 hours (lined bar). Results represent the mean ± SEM of 4 (A and B) to 6 (D) individual rats. One way ANOVA was performed in C (p<0.0001; F_2, 11_ = 30.90, n = 4–6) followed by Newman-Keuls multiple comparison test (***p≤0.001 vs control. +++p≤0.001 vs vehicle).

Bcl2 immunostaining was also evaluated in the striatum and SNpc of 6-OHDA-lesioned rats. In the lesioned striatum of IP-vehicle and IP-LGF treated rats the number of Bcl2-positive cells was 1.2-fold higher than in the naïve side of the structure [734±59 (n = 9) and 883±78 (n = 9) Bcl2-positive cells/mm^2^ in the naïve and lesioned striatum, respectively. t = 4.916, df = 8, p = 0.0012 vs the lesioned mesencephalon of IP-vehicle]**.** In the SNpc, Bcl2 immunoreactivity was not affected by the lesion with the neurotoxin (Fig. 9A and B), but 48 hours after LGF administration the number of Bcl2-positive cells was up-regulated in the lesioned side as compared with the naïve and lesioned SNpc of IP-vehicle treated rats (Fig. 9C and D).

## Discussion

In the present study, we show that the intraperitoneal administration of Liver Growth Factor (IP-LGF) significantly increases TH-positive innervation, TH and DAT protein expression, and DA levels in the striatum of 6-OHDA-lesioned rats. Moreover, IP-LGF treatment partially protects TH-positive neurons in the SNpc, and ameliorates motor behavior in these animals. Our results also point out that IP-LGF stimulates the phosphorylation of ERK1/2 and CREB in the DA-depleted striatum, and modulates the expression of Bcl2 and Akt in the striatum and mesencephalon of 6-OHDA-lesioned rats. The results also suggest the possible role of activated microglia in these LGF actions.

We reported previously that intracerebral administration of LGF stimulates TH-positive innervation in the striatum and increases neurogenesis in the subventricular zone of 6-OHDA-lesioned rats [10], [11]. Here we show that IP-LGF was able to potentiate TH-positive innervation and TH-protein expression in the DA-depleted striatum**.** In addition, IP-LGF partially restored DA levels in the striatum and protected TH-positive neurons in the SNpc. The fact that IP-LGF significantly reduces apomorphine-induced rotational behavior and improves motor performance, as assessed by the rotarod test, suggests that LGF modulates the functionality of the DA system in 6-OHDA-lesioned rats. In this respect, we should mention that DAT is a protein located on dopaminergic nerve terminals that is involved in DA uptake and is used as a marker for the evaluation of the integrity of the DA system [38]–[40]. As shown here, IP-LGF prevented the decrease in DAT protein expression and immunoreactivity promoted by 6-OHDA in the DA-depleted striatum. Moreover, LGF activated DAT trafficking in DA neurons because in those animals chronically treated with the factor, DAT immunostaining was observed in the TH-positive terminals. However, in the naïve and DA-depleted striatum, DAT immunoreactivity was confined to small spots that represent neuronal cell bodies.

We have recently demonstrated that LGF increases the survival of neural precursors when grafted in the DA depleted striatum of 6-OHDA-lesioned rats [12]. Similarly, IP-LGF may protect nigral DAergic neurons from cell death, as was reported for glial-derived neurotrophic factor (GDNF) and VEGF in 6-OHDA-lesioned rats [41]–[43]. Present results support this possibility, because during the time period between 6 and 13 weeks post-lesion the number of surviving TH-positive nigral neurons was further reduced in rats receiving vehicle, whereas it was maintained in IP-LGF treated animals. Besides having a protective effect, IP-LGF could promote the sprouting of DA terminals in the striatum, as was reported for VEGF [43], and for LGF when directly applied in the DA denervated striatum of 6-OHDA-lesioned rats (10).

An important issue was to determine the target cell and the molecular effectors that mediate the LGF-induced effects in 6-OHDA-lesioned rats. Our previous studies proposed that activated microglia could mediate the neuroregenerative and neurogenic effects promoted by LGF when this factor was delivered directly into the brain [10], [11]. As shown here. As shown here, the acute administration of LGF potentiated the expression of several markers that are associated with the activation of microglia. Thus, a single dose of IP-LGF stimulated GLUT-5 and OX6 protein expression in the denervated striatum. Moreover, IP-LGF increased proliferation, as measured by PCNA levels, and the number of OX6-positive cells that were proliferating, which is part of the activation response of microglia. Because the chronic administration of LGF restored the abovementioned parameters to levels similar to those observed in the naïve striatum, we propose that activation of microglia is an early and transitory event in the action of LGF. Activated microglia have been associated with the pathogenesis of several neurodegenerative diseases including PD [44]. Additionally, these cells may play a key role in the development and regeneration of the central nervous system through the release of trophic factors and extracellular matrix molecules [45]–[50]. In this respect, the available evidence suggests that the secretion of GDNF and brain-derived neurotrophic factor (BDNF) by activated microglia and/or macrophages induces the sprouting of DA fibers in the injured striatum [51]–[54]. We have not analyzed whether LGF-activated microglia are able to synthesize and release these neurotrophins, but we found that a single injection of LGF stimulated TNF-alpha protein expression and immunoreactivity in the DA-depleted striatum of 6-OHDA-lesioned rats. Because TNF-alpha-positive cells in the striatal parenchyma co-expressed OX6 and exhibited the morphology of microglia, we can argue that the acute administration of LGF directly stimulates this cell type to produce this cytokine. As a matter of fact, LGF induces TNF-alpha release in human monocytes (Dr. Díaz Gil, personal communication), and microglia originate from the invasion of monocytes during early development [55].

In the damaged liver, LGF regenerative activity is mediated by a local and transitory up-regulation of TNF-alpha mRNA and protein expression [13]. Similarly, TNF-alpha produced by microglia and/or monocytes could be responsible for the neurotrophic effect of LGF on DA neurons. Several reports have indicated a possible role of TNF-alpha signaling in neuroprotection [16], [17], [56], [57] and neurite outgrowth [18]. In this respect, it has been proposed that TNF-alpha released by microglia promotes neuronal survival by inducing the expression of neurotrophins and anti-apoptotic proteins in neurons and astrocytes [16]. Bcl2 is an anti-apoptotic protein involved in the survival of neural cells [29], [58]–[60], and its production is stimulated after the infusion of LGF into rat brain [12]. Interestingly, IP-LGF significantly raised its expression in the striatum and mesencephalon of 6-OHDA-lesioned rats. Since the administration of the factor did not affect the levels of the pro-apoptotic protein Bax, we can argue that LGF probably strengthens the mechanisms that regulate the viability of DA neurons. On the other hand, LGF increased GFAP protein expression and promoted cell body hypertrophy of GFAP-positive cells in the striatum, both of which are features of reactive astrocytes that are able to synthesize and release neurotrophins [61]–[63].

In spite of the quick increase in microglial activation and TNF-alpha expression shortly after a unique IP injection of LGF (acute treatment), a chronic administration of LGF generated a significant reduction of OX6 protein expression and of TNF-alpha levels in the lesioned mesencephalon and in the DA-depleted striatum, respectively. Therefore, our data suggest that a chronic treatment of LGF has anti-inflammatory effects on 6-OHDA neurotoxicity, which is in keeping with our previous studies, demonstrating LGF anti-inflammatory activity *in vivo* [7], [64] and anti-oxidant activity both in *in vitro* and *in vivo* systems [65], [66]. Although LGF treatment induced a transient increase in microglia and TNF-alpha levels, present data show that chronic administration of LGF reduced neuroinflamation and partially protects DA neurons from 6-OHDA neurotoxicity, which is consistent with the beneficial effects of several treatments (paroxetine, ketogenic diet) that are due to an inhibition of microglial activation and a reduced expression of TNF-alpha in an experimental model of PD [67], [68].

Thus far, nothing is known regarding the possible signal transduction pathways involved in LGF actions in brain. The interaction of many growth factors with their receptors stimulates the MAPK/ERK1/2 and PI3K/Akt signaling pathways, which are involved in neuroprotection and neuroregeneration [36], [69]–[72]. Here we report that a single IP-LGF injection elicits the phosphorylation of ERK1/2 in the DA-depleted striatum of 6-OHDA-lesioned rats. Because this effect was observed 24 hours after LGF treatment, it is difficult to determine whether it is due to a direct or to an indirect action of the factor. Two recent reports demonstrate the ability of albumin and bilirubin (LGF is an albumin–bilirubin complex) to activate the ERK1/2 signaling pathway in microglia and astrocytes [45], [73]. Moreover, our preliminary *in vitro* studies indicate that LGF activates the ERK1/2 signaling pathway in glial cells [74], so we would propose that ERK1/2 activation is due to a direct action of LGF on these cells. Nevertheless, we can not rule out the participation of TNF-alpha and/or neurotrophins in this LGF-mediated effect because both agents are able to stimulate the ERK1/2 signaling pathway in neural cells [30], [75], [76].

Our results also suggest that IP-LGF could modulate the activity of the PI3K/Akt signaling pathway, because a single IP-LGF injection significantly raised the phospho-Akt/Akt ratio in the DA-depleted striatum and damaged mesencephalon of 6-OHDA-lesioned rats. This signalling plays a critical role in the regulation of neuronal survival [77], [78], so it could be partially involved in the neuroprotective activity of LGF observed in this study. In this respect, we should mention that the expression of the anti-apoptototic protein Bcl2 can be regulated by the PI3K/Akt signaling pathway [35], [37], [79], and that Bcl2 levels were up-regulated in striatum and mesencephalon of 6-OHDA-lesioned rats treated with LGF. Nevertheless, we must also consider that this increase could be regulated by the activated ERK1/2 signaling pathway in the DA-depleted striatum, since Bcl2 levels are also regulated by these MAPKs [35], [80], and our previous *in vivo* studies show how the infusion of LGF into the striatum of 6-OHDA-lesioned rats stimulates ERK2 phosphorylation and up-regulates Bcl2 protein expression by 1.6- and 5-fold, respectively [12].

Present data show that IP-LGF promoted the phosphorylation in the striatum of CREB, a transcription factor involved in neural cell survival [81]–[84], and in axonal protection and regeneration [85]–[87]. Moreover, the factor regulates the expression of catecholamine biosynthetic enzymes and transporters [88]–[90] and, thus, its activation could be responsible for the increase in TH and DAT protein levels observed in the DA-depleted striatum of IP-LGF treated rats. From our study we can not determine whether CREB phosphorylation was mediated by the MAPK ERK1/2, and/or the PI3K/Akt signaling pathways because both were activated 24 hours after the administration of LGF, and maximal levels of phospho-CREB were observed 72 hours after the start of treatment. However, our *in vitro* studies show that LGF stimulates the phosphorylation of ERK1/2 and CREB in glia [74], suggesting that MAPK ERK1/2 signaling is responsible for the activation of CREB in these cells. Our *in vivo* unpublished observations also support the involvement of ERK1/2 in LGF-mediated CREB phosphorylation because the infusion of LGF into rat brain up-regulates phospho-ERK2 levels, and enhances phospho-CREB protein expression by 2-fold.

Finally, from our results we are not able to define if the herein observed effects of LGF are due to a direct or indirect action of the growth factor on the lesioned nervous tissue. A direct action would imply that LGF could cross the blood brain barrier. Several mechanisms may be involved in such a transport, as the caveola-mediated transcytosis occuring in the transendothelial transport of albumin and albumin-conjugated nutrients [91], [92]. Alternatively, LGF could bind to specific receptors present in the blood brain barrier. The receptors for the advanced glycation end products (RAGE) located in the endothelium [93] could be good candidates, since AGE products have similar biochemical properties to LGF, i.e. they are mostly albumins bound to glucose that changes albumin conformation [94]. Moreover, RAGE are present in astrocytes and microglia [95], and their stimulation elicits TNF-alpha release from these cells [96]. Further *in vivo* experiments with labeled LGF, measurements of brain stimulation and localization of LGF in the brain could clear up definitively whether LGF crosses the blood-brain barrier.

### Conclusions

In summary, our study shows that in 6-OHDA-lesioned rats intraperitoneal administration of LGF up-regulates striatal DA levels, and partially protects TH-positive terminals in the striatum and DA neurons of the SNpc from 6-OHDA neurotoxicity. LGF activity is plausibly mediated through the modulation of the MAPK ERK1/2 and PI3K/Akt signaling pathways, and the regulation of proteins that are critical for cell survival. Since this regeneration factor reduces apomorphine-induced rotational behavior and improves motor performance in these animals, we propose LGF as a novel factor that may be useful in the treatment of PD.
